# Shexiang Tongxin Dropping Pill Protects Against Chronic Heart Failure in Mice *via* Inhibiting the ERK/MAPK and TGF-β Signaling Pathways

**DOI:** 10.3389/fphar.2021.796354

**Published:** 2021-12-03

**Authors:** Shuying Zhang, Hanbing Liu, Qianqian Fang, Houhong He, Xiaoyan Lu, Yi Wang, Xiaohui Fan

**Affiliations:** ^1^ State Key Laboratory of Component-Based Chinese Medicine, Tianjin University of Traditional Chinese Medicine, Tianjin, China; ^2^ Inner Mongolia Conba Pharmaceutical Co., Ltd., Hohhot, China; ^3^ Zhejiang Conba Pharmaceutical Co., Ltd., Hangzhou, China; ^4^ Pharmaceutical Informatics Institute, College of Pharmaceutical Sciences, Zhejiang University, Hangzhou, China; ^5^ Westlake Laboratory of Life Sciences and Biomedicine, Hangzhou, China

**Keywords:** Shexiang Tongxin dripping pill, chronic heart failure, network pharmacology, whole-transcriptome sequencing, ERK/MAPK signaling pathway, TGF-β signaling pathway

## Abstract

**Background:** Chronic heart failure (CHF) is a major public health problem with high mortality and morbidity worldwide. Shexiang Tongxin Dropping Pill (STDP) is a widely used traditional Chinese medicine preparation for coronary heart disease and growing evidence proves that STDP exerts beneficial effects on CHF in the clinic. However, the molecular mechanism of the therapeutic effects of STDP on CHF remains largely unknown.

**Objective:** This study aimed to elucidate the mechanism of action of STDP against CHF by integrating network pharmacology analysis and whole-transcriptome sequencing.

**Methods:** First, the mouse model of CHF was established by the transverse aortic constriction (TAC) surgery, and the efficacy of STDP against CHF was evaluated by assessing the alterations in cardiac function, myocardial fibrosis, and cardiomyocyte hypertrophy with echocardiography, Masson’s trichrome staining, and wheat germ agglutinin staining. Next, a CHF disease network was constructed by integrating cardiovascular disease-related genes and the transcriptome sequencing data, which was used to explore the underlying mechanism of action of STDP. Then, the key targets involved in the effects of STDP on CHF were determined by network analysis algorithms, and pathway enrichment analysis was performed to these key genes. Finally, important targets in critical pathway were verified *in vivo*.

**Results:** STDP administration obviously improved cardiac function, relieved cardiomyocyte hypertrophy, and ameliorated myocardial fibrosis in CHF mice. Moreover, STDP significantly reversed the imbalanced genes that belong to the disease network of CHF in mice with TAC, and the number of genes with the reverse effect was 395. Pathway analysis of the crucial genes with recovery efficiency revealed that pathways related to fibrosis and energy metabolism were highly enriched, while TGF-β pathway and ERK/MAPK pathway were predicted to be significantly affected. Consistently, validation experiments confirmed that inhibiting ERK/MAPK and TGF-β signaling pathways *via* reduction of the phosphorylation level of Smad3 and ERK1/2 is the important mechanism of STDP against CHF.

**Conclusion:** Our data demonstrated that STDP can recover the imbalanced CHF network disturbed by the modeling of TAC through the multi-target and multi-pathway manner in mice, and the mechanisms are mainly related to inhibition of ERK/MAPK and TGF-β signaling pathways.

## Introduction

Chronic heart failure (CHF) is a clinical syndrome, caused by cardiac structural and functional abnormalities, leading to an imbalance between cardiac output and the metabolic demands of body (Ahmad et al., 2012). CHF induces a staggering burden to society with high mortality and morbidity. Approximately 2% of the global adult population suffer from this disease, and the 5-year mortality is reckoned at 45–60% (Mozaffarian et al., 2015; Metra and Teerlink, 2017; Virani et al., 2021). CHF is also the most frequent cardiovascular cause of hospitalization in over 65-year olds (Planelles-Herrero et al., 2017). Clinically, the main medical treatments for CHF are diuretics (Roubille et al., 2014), β-receptor blockers (Foody et al., 2002), and angiotensin converting enzyme inhibitor (Macdonald, 2015), which can rapidly improve the hemodynamic metrics of patients, but the prognosis and quality of life are still poor (Jia et al., 2020). More importantly, a prolonged treatment of these chemical drugs may cause extremely serious side effects, including hypotension and electrolyte depletion (Yancy et al., 2016), and a considerable and growing number of patients with CHF respond poorly to these drugs (Owan et al., 2006; McMurray, 2010). Therefore, new treatment options are urgently needed, particularly for the approaches targeting the variety of molecular processes involved in CHF with less side effects.

Shexiang Tongxin Dropping Pill (STDP) is a derivative of Zhibao mini-pills recorded in Taiping Huimin Hejiju Fang in the Song Dynast. It is made up of seven kinds of Chinese medicinal materials, including *Salvia miltiorrhiza* Bunge, *Moschus*, *Bovis Calculus Artifactus*, *Bufonis Venenum*, *Borneolum Syntheticum*, total ginsenoside of ginseng stems and leaves, and *Fel Ursi*. Forty-one constituents were identified in STDP and their types mainly involve bile acids, salvianolic acids, triterpene saponins, bufadienolides, and tanshinones (Chen et al., 2015). STDP has shown beneficial effects in the treatment of cardiovascular diseases in both basic and clinical researches (Lu et al., 2020; Ding et al., 2021). The primary indication of STDP is for coronary heart disease, especially for the stable angina pectoris. The rationale behind this indication lies in STDP clinically protects vascular endothelial cells, reduces myocardial fibrosis, and improves coronary microcirculation (Ning, 2012; Wang et al., 2019), which are also beneficial to other cardiovascular diseases. Consistently, growing evidence shows that STDP administration can promote cardiac function in the patients with CHF (Hua et al., 2011), although CHF is not an indication for STDP. Additionally, multiple clinical studies have also reported that the combination of STDP and conventional therapy has significant efficacy in the treatment of CHF by improving cardiac circulatory function and suppressing inflammatory cytokines in patients (Quan et al., 2021). Nevertheless, the underlying mechanism of STDP in the treatment of CHF had rarely been reported, which is important for clinical application of this drug.

Owing to the complexity of the chemical composition of STDP, it is difficult to elucidate its multi-target and multi-pathway mechanisms by relying solely on the traditional pharmacological methods with the model of “one target, one drug”. Fortunately, the emergence of network pharmacology provides a novel perspective for interpreting the mechanisms of action of Traditional Chinese Medicine (TCM) prescriptions. Network pharmacology integrates system biology, bioinformatics, and polypharmacology, which coincides with the holistic characteristics of TCM (Gao et al., 2020; Huang et al., 2020). Currently, this approach is also increasingly being applied to repurpose already approved drugs and explore their new therapeutic potential (Kibble et al., 2015). However, one of the disadvantages is that network pharmacology cannot distinguish whether the genes were upregulated or downregulated by drug treatment, whereas, transcriptome sequencing can remedy this shortcoming (Wu et al., 2019; Zhu et al., 2021). Therefore, in the present study, integrating network pharmacology with whole-transcriptome sequencing analysis was conducted to explore the multi-target and molecular mechanisms of STDP against CHF in mice. The predicted mechanisms were also verified *in vivo*.

## Materials and Methods

### Drug and HPLC Analysis

STDP (Lot No. 170204) was provided by Inner Mongolia Kangenbei Pharmacy Co., Ltd. (Erdos, Inner Mongolia, China; CFDA Med drug permit NO. Z20080018). STDP includes *Salvia miltiorrhiza* Bunge (Lamiaceae; *Salviae miltiorrhizae* radix *et* rhizoma), total ginsenoside of ginseng stems and leaves (from *Panax ginseng* c. a. Meyer, Araliaceae; *Ginseng Radix et Rhizoma*), *Moschus* (the dried secretion of musk sac of adult male *Moschus berezovskii* Flerov*, Moschus sifanicus* Przewals, or *Moschus moschiferus* Linnaeus), *Bovis Calculus Artifactus* (prepared with powder of cow bile, hyodeoxycholic acid, cholic acid, taurine, cholesterol, bilirubin, trace elements, etc), *Bufonis Venenum* (the dried secretion of *Bufo bufo gargarizans* cantor or *Bufo melanostitus* Schneider), *Borneolum Syntheticum* (the weight of synthetic crystal dominated by borneol not less than 55%), and *Fel Ursi* (the dried bile in the gallbladder of *Selenarctos thibetanus* G. Cuvier, or *Ursus arctos* Linnaeus). The whole production process of STDP, from the verification of raw materials to the final product, totally complied with the provisions of Chinese Pharmacopoeia (2020 Edition). The specific processing steps of STDP were as follows: *Salvia miltiorrhiza* Bunge was water-extraced, followed by multi-step of alcohol precipitation to remove the impurities, and then concentrated to extractum. Meanwhile, the ethanol extract of *Bufonis Venenum* was prepared, the impurities were removed by salting-out method, and the sample was dried under reduced pressure. Moisten *Bovis Calculus Artifactus*, total ginsenoside of ginseng stems and leaves, *Salvia miltiorrhiza* Bunge extract, and *Fel Ursi* were soaked with pure water, while *Bufonis Venenum* extract and *Borneolum Syntheticum* were soaked with ethanol. After that, they were all added into the molten polyethylene glycol 6000 together with *Moschus*, red iron oxide, black iron oxide, and polysorbate 80, mixed evenly and prepared into dripping pills. HPLC analysis of STDP was performed (Supplementary Figure S1). The main constituents in STDP were detected including danshensu, protocatechualdehyde, salvianolic acid D, salvianolic acid B, salvianolic acid A, taurodeoxycholic acid, cinobufotalin, bufalin, cinobufagin, and resibufogenin. The content of salvianolic acid B, resibufogenin, bufalin, and cinobufagin in STDP were 0.037, 0.013, 0.011 and 0.029 mg per pill, respectively.

### Animals

Male C57BL/6 mice (6–8 weeks of age, 22–25 g) were purchased from Shanghai SLAC Laboratory Animal Co., Ltd. (Shanghai, China) and housed in a temperature (25 ± 1°C) and humidity-controlled (50 ± 10%) room under a 12-h light-dark cycle. Mice were free accessed to standard diet and clean water. All animal experiments were carried out according to the guidelines of the Animal Care and Use Committee of Zhejiang University School of Medicine.

### Model of CHF and Drug Treatment

The mouse model of CHF was established by transverse aortic constriction (TAC) as previously described (Arany et al., 2006; deAlmeida et al., 2010; Liu et al., 2019). This is a widely used murine model of pressure overload-induced heart failure. After the mice were anesthetized by intraperitoneal injection of 0.3% pentobarbital sodium at a dose of 75 mg/kg, skin was cut longitudinally above the sternum and a partial thoracotomy to the second rib is performed in the mid upper thorax. The thymus and adipose tissue were then retracted using micro forceps to expose the transverse aorta, which is located between the brachiocephalic and left common carotid arteries. A 6–0 nylon suture was tied loosely around the transverse aorta with a single knot, and a pre-sterilized, blunt-end needle (26-G) was then placed within the knot, alongside the aorta. Then, the knot was tightened fully, and the blunt-end needle was removed. The incision was closed in layers with a 6–0 nylon suture. After the operation, each mouse was intraperitoneally injected with 0.2 ml furosemide injection (Jilin Huamu Animal Health Products Co. Ltd., Changchun, China) to avoid the occurrence of acute heart failure, and the wound was gently daubed with penicillin sodium (CSPC NBP Pharmaceutical Company, Shijiazhuang, China). Mice in the Sham group underwent a same procedure except for the ligation of the aorta.

One week after the operation, the surviving mice were randomly divided into three groups: 1) sham-operated group (Sham, intragastrically administered 1% sodium carboxymethyl cellulose at 0.1 ml/10 g body weight); 2) model group (Model, intragastrically administered 1% sodium carboxymethyl cellulose at 0.1 ml/10 g bw); 3) STDP treated group (STDP, intragastrically administered STDP at 86 mg/kg bw). The drugs were administered once a day for 6 weeks. The dosage of STDP was approximately twice the equivalent dose of STDP used in the clinic for adults (3.5 mg/kg bw per day), which is converted by normalization of body surface area.

### Echocardiographic Evaluation of Cardiac Function

After chest hair was removed with depilatory cream, the mice were anesthetized with isoflurane (Jiangsu Hengfengqiang Biotechnology Co., Ltd., Nanjing, China) and fixed on the operating plate in the supine position. Subsequently, transthoracic echocardiographic images from all groups of unconscious mice were acquired using a Vevo 1100 ultra-high resolution small animal imaging ultrasound system (Fujifilm VisualSonics, Toronto, ON, Canada) with an ultrasound probe. The cardiac function indexes, including the cardiac output (CO), the left ventricular ejection fraction (LVEF), and the left ventricular fractional shortening (LVFS), were calculated by the Vevo 1100 software.

### Histological Analysis

Mouse hearts were harvested and fixed in 4% paraformaldehyde. After paraffin embedding and slicing, the heart sections were subjected to Masson’s trichrome staining to detect interstitial fibrosis. Meanwhile, a part of sections was stained with FITC-conjugated wheat germ agglutinin (WGA for short, Sigma-Aldrich, St. Louis, MO, United States) for determination of cardiomyocyte size. Digital images were randomly selected and captured using inverted fluorescence microscope (Nikon Eclipse TI-SR, Japan) for each sample.

### Transcriptome Analysis

First, total RNA from heart tissue was extracted with the TRIzol™ Reagent (Thermo Fisher Scientific, MA, United States), and the purity and integrity of RNA were detected by agarose gel electrophoresis. Second, sequencing libraries were generated using the Illumina NEBNext^®^ Ultra™ RNA Library Prep Kit (NEB, United States) according to the protocols recommended by the manufacturer. Briefly, the mRNA was randomly interrupted and reverse transcription was performed to synthesize double-stranded cDNA, which was purified (∼200bp) by the AMPure XP beads after ligation of adaptors to obtain the sequencing library. Third, the content of DNA was detected by a Qubit 2.0 Fluorometer (Invitrogen, Carlsbad, CA, United States), and the quality of the library DNA integrity was assessed using an Agilent 2,100 Bioanalyzer (CA, United States). Last, the qualified samples were sequenced on an illumine HiSeq X Ten platform by Novogene (Beijing, China).

The quality of the raw sequencing data was controlled and the acquired raw image files were converted to sequential reads by CASAVA (Illumina, Inc) and stored in FASTQ format. Next, genomic alignment and quantification of gene expression levels were performed using Hisat2 v2.0.5 and featureCounts v1.5.0. Subsequently, DESeq2 (v1.16.1) package was used to analyze the gene expression difference between two groups. The *p* value and the fold change were obtained to select the differentially expressed genes (DEGs) between Sham and Model or Model and STDP groups. All the RNA sequencing data have been uploaded to Gene Expression Omnibus (GEO) database of National Center for Biotechnology Information (NCBI) under accession number GSE185631.

### Construction and Analysis of CHF Disease Network

#### Construction and Visualization of CHF Disease Network

Based on the CHD@ZJU3.0 (http://tcm.zju.edu.cn/chd/) established previously by our group (Wu et al., 2013), cardiovascular disease-related genes of mice dataset were extracted. An intersection between these disease-related genes and sequenced genes detected in the transcriptome analysis was generated using the Dplyr tool and then used to construct a CHF disease network. Furthermore, the protein-protein interactions (PPI) analysis was performed in the String database (https://string-db.org/) and visualized using the CytoscapeV3.8.0 software. Pathway analysis was carried out to the overlapping genes using the Kyoto Encyclopedia of Genes and Genomes (KEGG) database (https://www.kegg.jp/) in order to select the more important pathways in CHF.

#### Evaluation of Key Genes in CHF Disease Network

We further evaluated the key nodes (core genes) in the CHF disease network based on our previous proposed NTRA algorithm (Liao et al., 2018). This approach integrated the transcriptomics and topology attributes of nodes to achieve priority ranking, and then get the function of the key nodes in the network. In short, topological Rank (RankT) was composed of relative ranks of the Betweeness (RankB) and the Degree (RankD), calculated by Network Analyzer tool in Cytoscape V3.8.0 software (Eq. 1), while betweenness and degree meant the significance of nodes in the overall biological network (Bozhilova et al., 2019). Transcriptome Rank (RankR) was consisted of *p* value rank (RankP) and Fold Change rank (RankF) (Eq. 2), in which genes with smaller *p* value rank and larger Fold Change indicated more vital genes. Additionally, the NTRA Rank (Rank) was calculated by combining RankT and RankR (Eq. 3), and the higher NTRA rank represented the more crucial the gene was in the CHF disease network.

RankT=R(RankB+RankD) (1)

RankR=R(RankF+RankP) (2)

Rank=R(RankT+RankR) (3)

#### Evaluation the Efficacy of STDP Against CHF by Disease Network

The efficacy of STDP against CHF in mice was evaluated according to the Efficiency of Recovery regulation (EoR, Eq. 5) proposed in our previous study (Wu et al., 2014). In this method, the recovery level (RL) was computed by comparing the gene expression changes between STDP/Model and Sham/Model (Eq. 4) and the EOR value was subsequently obtained based on Eq. 5. The maximum value of EOR was 100%, indicating that STDP treatment completely corrected the gene disorder induced by TAC modeling, while EoR <0 indicated no recovery effect.

$$RL=\frac{Fold\;Change\;\left(STDP/Model\right)}{Fold\;Change\;\left(Sham/Model\right)}\;$$ (4)

EoR=100%−|100%−RL| (5)

#### Pathway Enrichment Analysis

Genes ranked in the top 50% in NTRA data with EOR >0 were selected for pathway enrichment analysis, which was performed by using Ingenuity Pathways Analysis (IPA) and via Metascape (https://metascape.org/gp/index.html), respectively.

### Quantitative Real-Time Polymerase Chain Reaction (qRT-PCR)

The qRT-PCR was conducted to verify the accuracy of the transcriptome sequencing data, which was specifically operated as below. The RNA content of mouse heart tissue was measured by a Nanodrop 2000 ultra-micro spectrophotometer (Thermo Fisher Waltham, MA, United States), and the same amount of RNA samples from three mice in the same group were mixed and represented as a sample for this group. The RNA samples were subjected to reverse transcription according to the instruction of the Reverse Transcription Kit (QIAGEN, Hilden, Germany). The resulting cDNA was used for real-time PCR on a CFX-Touch 96 Real-Time PCR system (Bio-Rad Laboratories, Hercules, CA) with the Hieff UNICON^®^ qPCR SYBR Green Master Mix (Shanghai, China) and specific primers (Supplementary Table S1). The relative gene expression level was calculated by 2^−ΔΔCt^ method. The housekeeping gene *Actb* was used to normalize the data, and the experiment was repeated three times.

### Western Blot Analysis

Heart tissues (20 mg per/simple) were homogenized in RIPA buffer (600 μL) with 1% PMSF. The protein concentration was determine using a BCA Protein Assay kit (Thermo Fisher Waltham, MA, United States), and the same amount of protein samples of three mice in the same group were mixed and represented as the sample of this group. Then, protein samples were electrophoresed by 10% TGX Stain-Free polyacrylamide gels (Bio-Rad, Hercules, CA, United States) and transferred to polyvinylidene difluoride membranes (Merck Millipore). After blocking, membranes were incubated with the following primary antibodies at 4°C: ERK1/2 (9102S, 1:500, Cell Signaling Technology, Beverly, MA, United States), *p*-ERK1/2 (8544S, 1:500, Cell Signaling Technology, Beverly, MA, United States), Smad3 (AF1501, 1:500, Beyotime Biotechnology, Shanghai, China), *p*-Smad3 (AF1759, 1:500, Beyotime Biotechnology, Shanghai, China), and GAPDH (AF1186, 1:500, Beyotime Biotechnology, Shanghai, China). After overnight incubation, membranes were washed and hybridized with horseradish peroxidase (HRP)-conjugated secondary antibodies for 1h, including the anti-mouse (A0216, 1:2000, Beyotime Biotechnology, Shanghai, China) and anti-rabbit (A0208, 1:2000, Beyotime Biotechnology, Shanghai, China) HRP-conjugated secondary antibodies. Finally, the blots were detected by the enhanced chemiluminescent substrate reagent and imaged using a ChemiDoc™ Imaging System (Bio-Rad, Hercules, CA, United States). Relative protein levels were counted using the Bio-Rad Image Lab software and GAPDH was used as the internal standard.

### Statistical Analysis

All data are expressed as the mean ± standard deviation (SD). GraphPad Prism eight software was used to analyze, graph, and present data. Statistical differences were examined by one-way ANOVA followed by Dunnett’s multiple comparison test; differences were considered significant at *p* values less than 0.05.

## Results

### STDP Ameliorated Cardiac Disfunction in CHF Mice

**FIGURE 1:**
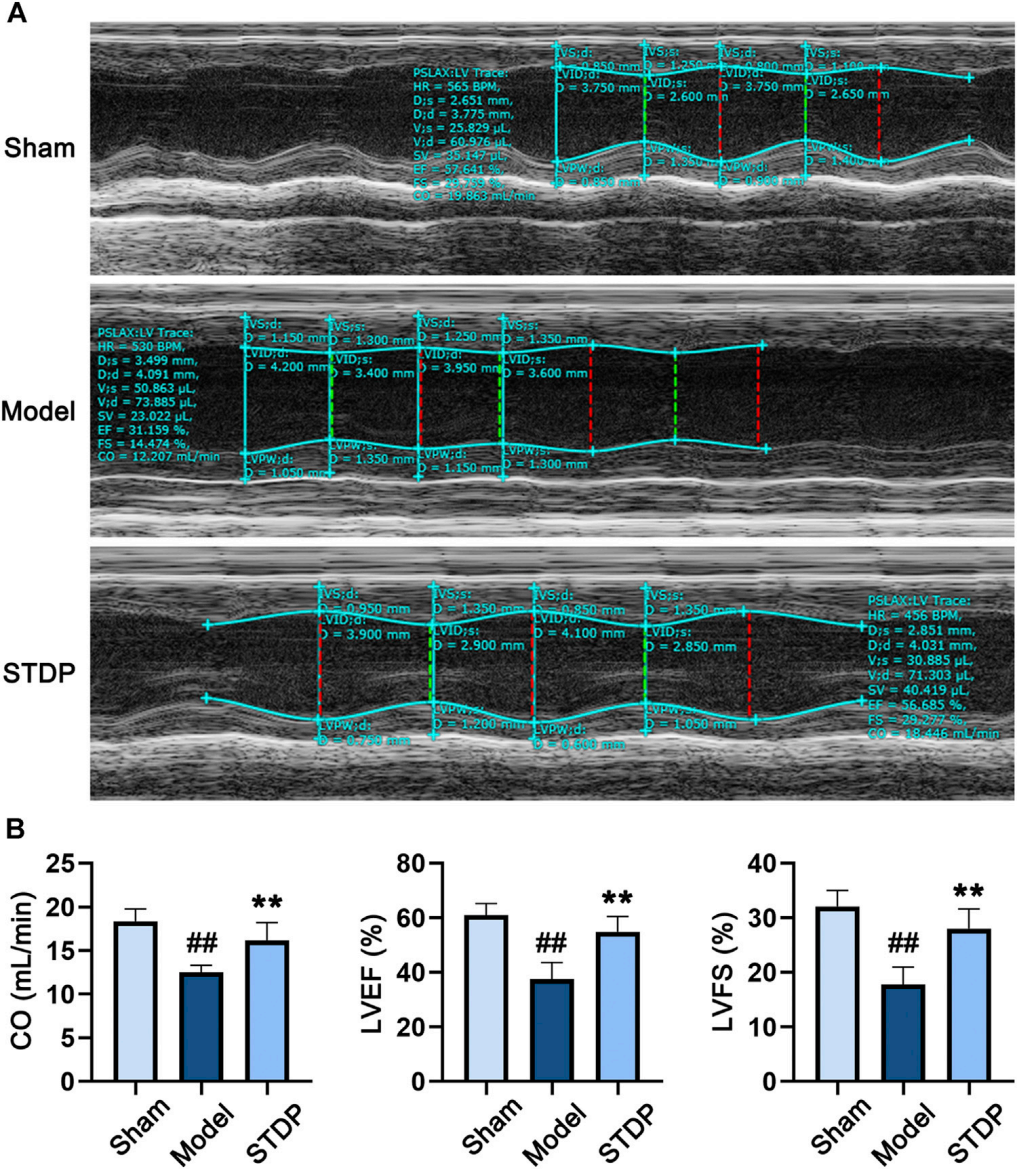
STDP relieves TAC-induced cardiac dysfunction in CHF mice. **(A)** Representative echocardiograms after 6 weeks of STDP treatment (86 mg/kg/d). **(B)** The cardiac function indexes of mice were quantified by the Vevo 1100 software. ^##^ *p* < 0.01 versus the Sham group; ^**^ *p* < 0.01 versus the Model group; n = 7.

First, we evaluated the effect of STDP on CHF mice after 6 weeks of drug treatment. Critical cardiac parameters were analyzed by echocardiography, including CO, LVEF, and LVFS. As expected, compared with the Sham group, TAC surgery (Model) resulted in significant diminution in CO, LVEF, and LVFS (Figures 1A,B), indicating the pathological change of left ventricle. After treating CHF mice with STDP, these cardiac function indices were dramatically increased compared with the Model group, suggesting that STDP could attenuates pathological cardiac dysfunction in a mouse model of CHF.

### STDP Relieved TAC-Induced Cardiomyocyte Hypertrophy in Mice

**FIGURE 2:**
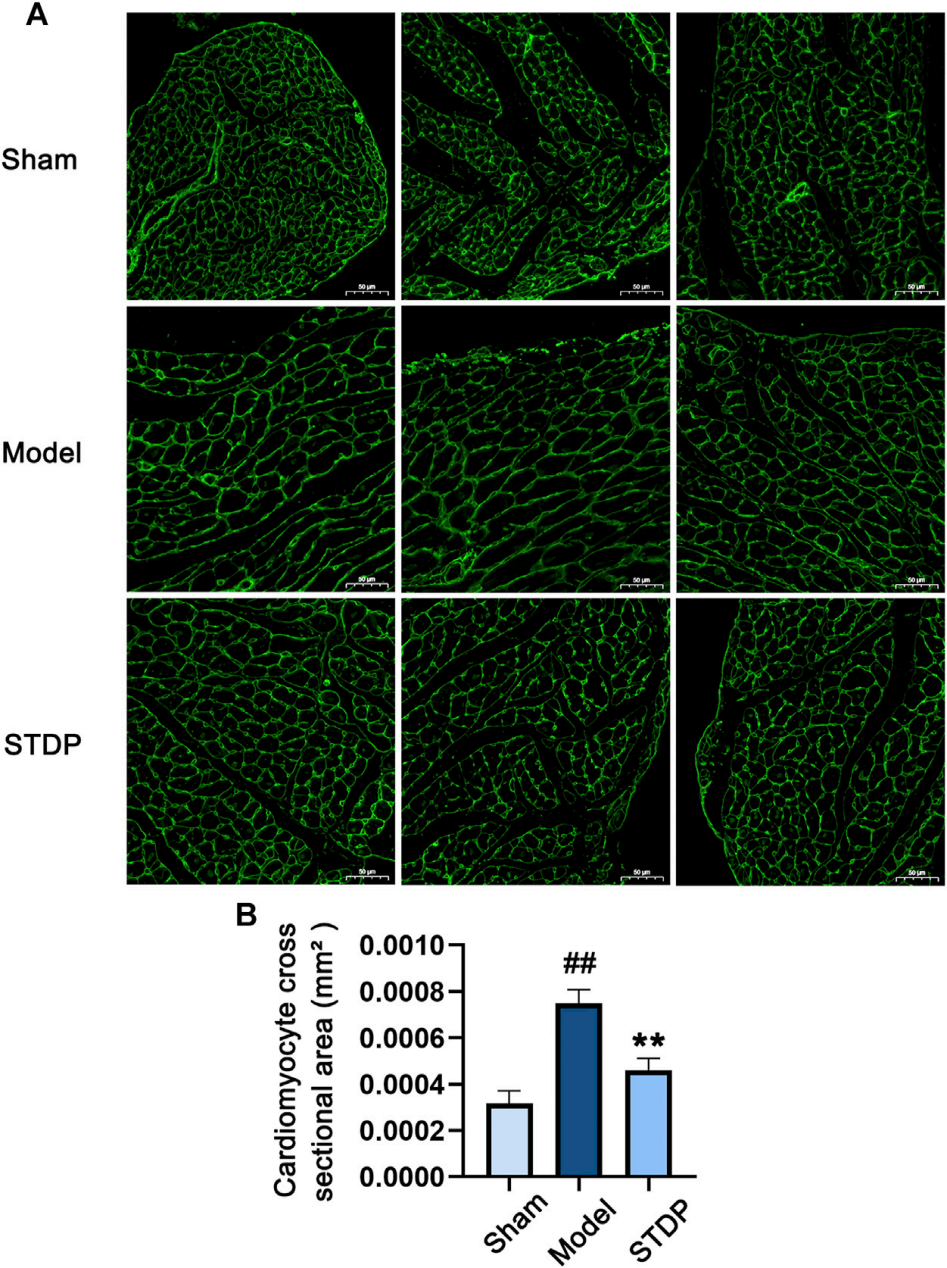
STDP reduces cardiomyocyte hypertrophy in CHF mice. **(A)** Representative images of mouse left ventricular sections stained with WGA and photographed with fluorescence microscope (magnification, ×400). Bars = 50 μm. **(B)** Statistics of cardiomyocyte cross sectional area in mice. ^##^ *p* < 0.01 versus the Sham group; ^**^ *p* < 0.01 versus the Model group; n = 3.

Cardiomyocyte hypertrophy is an important pathological link in the process of CHF (Xu et al., 2015; Rifai et al., 2020). Thus, we further detected the protective effect of SDPT on TAC-induced cardiomyocyte hypertrophy by WGA staining. Notably, while TAC surgery significantly increased cardiomyocyte cross-sectional area (Figures 2A,B), this structural change was significantly attenuated in STDP-treated mice, indicating the beneficial effect of STDP on the reduction of cardiomyocyte hypertrophy in CHF mice.

### STDP Ameliorated Cardiac Fibrosis in CHF Mice

**FIGURE 3:**
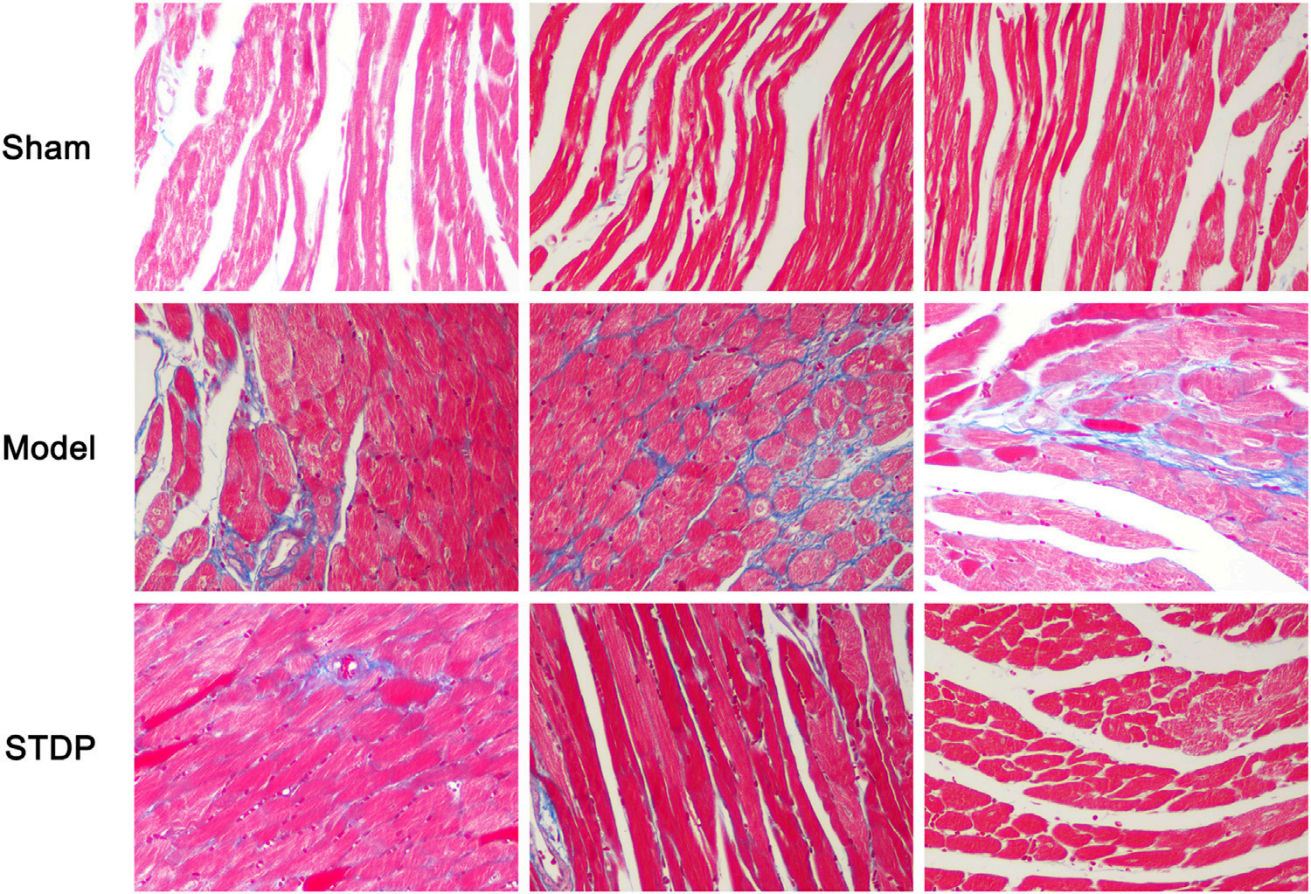
STDP attenuates myocardial fibrosis in CHF mice. After 6 weeks of STDP treatment, representative images of mouse left ventricular sections stained with Masson’s trichrome and photographed with fluorescence microscope (magnification, ×400). Bars = 50 µm. n = 3. Collagen fibers were stained blue and myocytes were stained red, reflecting the degree of myocardial fibrosis.

As myocardial interstitial fibrosis is a critical contributor to the left ventricular dysfunction and cardiac hypertrophy leading to the development of heart failure (Gonzalez et al., 2018; Li et al., 2020a), we used Masson’s trichrome staining to detect the degree of myocardial fibrosis in the CHF mice after STDP administration. The results showed that Sham group displayed normal morphology in the myocardial tissue, while mice in the Model group had marked interstitial fibrosis in the left ventricular myocardial regions (Figure 3). In contrast, STDP treatment obviously ameliorated myocardial interstitial fibrosis in the mice subjected to TAC, implying that STDP reduces cardiac fibrosis in CHF mice.

### STDP Recovered the Gene Expression Disturbed by TAC in CHF Disease Network

To further explore the potential pharmacological mechanism of STDP against CHF, we adopted a strategy of combining network pharmacology and transcriptomics analyses. First, the whole-transcriptome of mouse heart tissue samples was sequenced, and qRT-PCR was performed to verify the accuracy of sequencing data. The results of qRT-PCR highly corresponded with primary RNA-sequencing data (Figure 6A). Namely, both detection methods showed that the expression levels of selected genes (*Tgfb3*, *Tgfbr1*, *Mybphl1*, and *Bmp10*) were upregulated in the Model group, and this effect was reversed after treatment with STDP.

**FIGURE 4:**
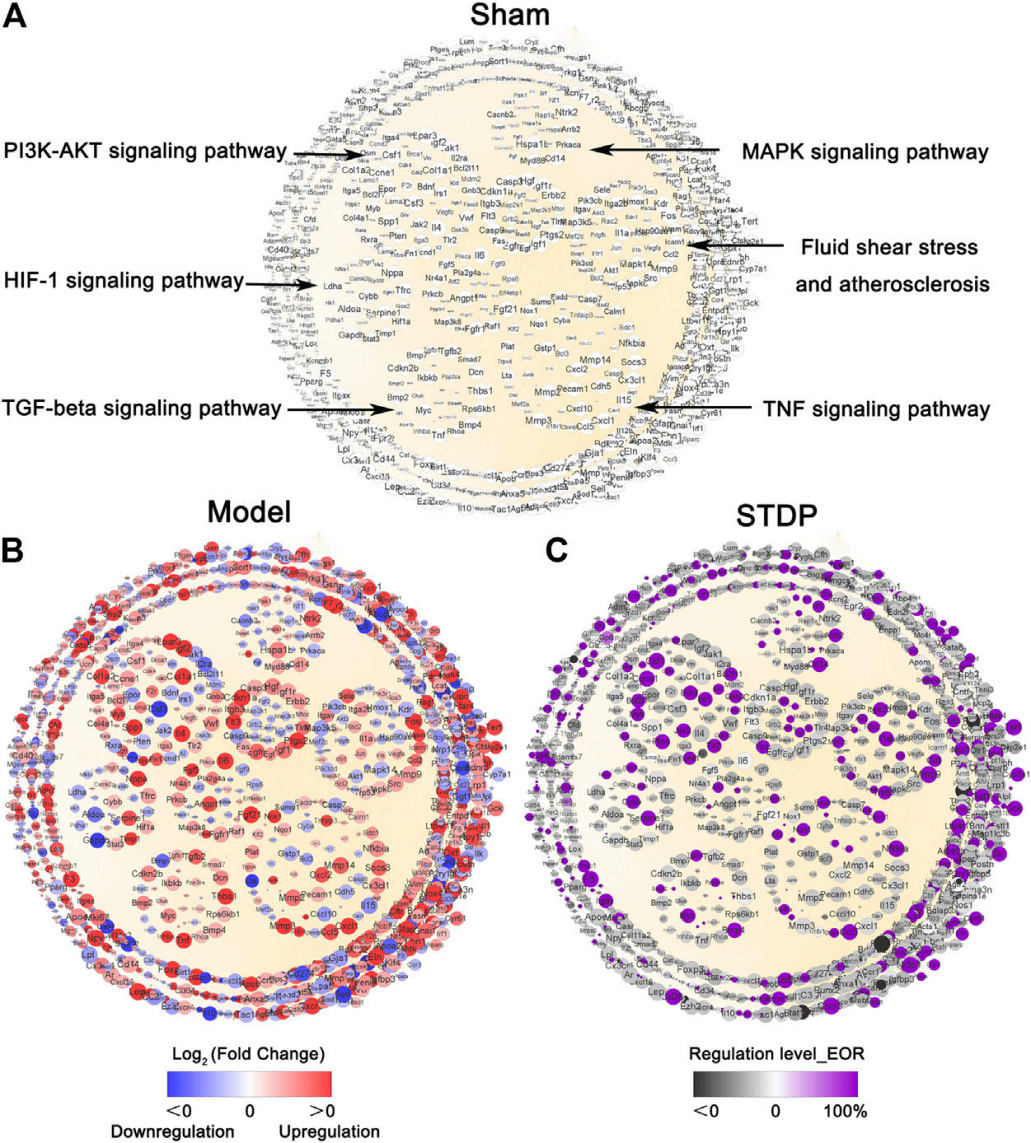
STDP recovers imbalanced CHF network disturbed by TAC modeling. **(A)** CHF disease network in the Sham group. Each node represents a gene, and each edge represents the relationship between two genes based on the protein-protein interaction. The size of node represents NTRA rank, and the circles in the center of the network indicate the crucial pathways. **(B)** TAC modeling disrupted CHF network. The red color of the genes represents log2 Fold Change (Model/Sham) > 0, implying upregulated expression after TAC modeling; whereas blue indicate log2 Fold Change (Model/Sham) < 0, meaning downregulated gene expression by TAC. The gradation of the color indicates the degree of gene upregulation and downregulation. **(C)** The recovery regulation of STDP on CHF network. The purple color of the gene indicates that the recovery efficiency EoR >0. It means that STDP treatment shows a recovery effect on the gene. The black color indicates no effect. The gradation of the color represents the efficiency of recovery regulation.

Next, CHF network was constructed with the overlapping parts (1636 genes) of the 54,532 sequenced genes and the 1643 genes extracted from our previous CHF database. In the process of network construction, 123 genes did not find any interaction relationship based on String 11.0 database, the resulting CHF disease network contained 1,513 nodes and 48,353 edges. Cytoscape software (V3.8.0) was applied to visualize this network (Figure 4A). Among this network, the nodes represent the genes involved in the pathogenesis of CHF, and the edges indicate the relationship between these genes which was based on the protein-protein interactions. This network implied that CHF disease is a complex disease involving multiple targets and pathways. Furthermore, significantly enriched pathways were generated based on KEGG analysis and gathered in the middle of the network, and these pathways are closely related to the occurrence and development of CHF. Then, the grave implications of TAC modeling were visualized through the gene expression changes between Sham and Model groups. Specifically, compared with Sham group, the red node indicates Log_2_ (Fold Change) > 0 and implies upregulation of the gene expression by TAC modeling, while the blue node represents Log_2_ (Fold Change) < 0 and means downregulation of the gene expression after TAC surgery. Therefore, the multicolor network indicated an extremely obvious disorder of gene expression in the Model group, implying that CHF model conspicuously damaged the cardiac function (Figure 4B). The critical biological pathways disturbed by TAC modeling were the TNF signaling pathway, MAPK signaling pathway, TGF-β signaling pathway, and PI3K-Akt signaling pathway. Moreover, the nodes with purple color in Figure 4C represent the recovery genes after STDP treatment, marked by EOR >0. As expected, 395 genes presented a distinctly recovery trend after STDP treatment, among which 77.7% nodes exhibited more than 80% efficiency of recovery regulation, suggesting that STDP might play an anti-CHF role by regulating these genes.

### STDP Attenuated TAC-Induced CHF in Mice Mainly by Energy Metabolism and Fibrotic Signaling Pathway

**FIGURE 5:**
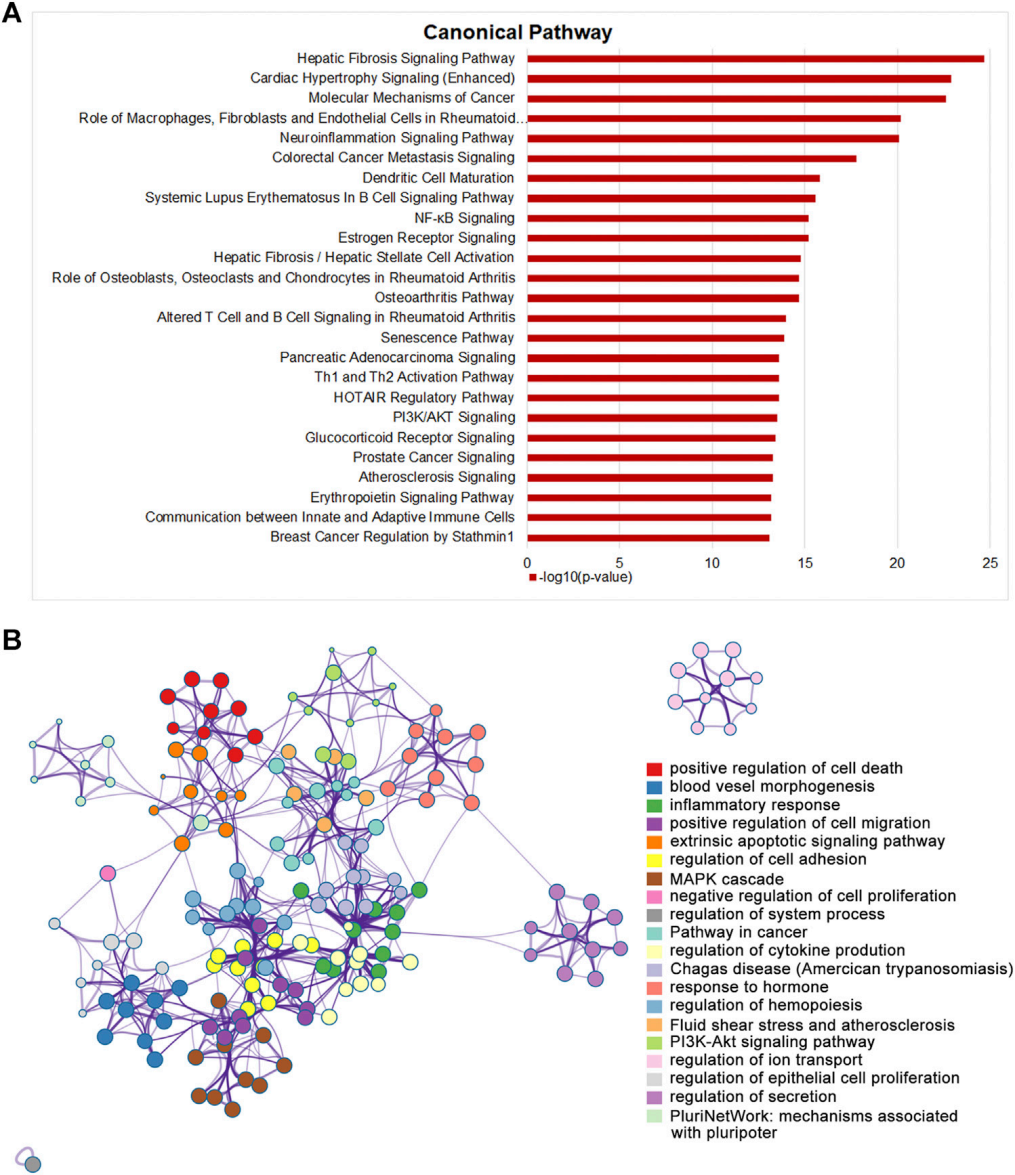
STDP improves TAC-induced CHF in mice mainly by energy metabolism and fibrotic signaling pathway. **(A)** The top 25 classical pathways significantly enriched after STDP treatment by IPA. **(B)** A network showing interactions among the gene clusters with significant enrichment in molecular functions and biological processes using Metascape.

Integrating both topology and transcriptomics parameters for comprehensive exploration the mechanisms of STDP against CHF, 165 genes that were ranked in the top 50% in NTRA data with EOR >0 was used to perform pathway enrichment analysis by IPA software. As the results, a total of 419 pathways were obtained, among which the top 25 pathways were shown in Figure 5A. It is worth noting that pathways related to the occurrence and development of CHF were obviously enriched, including fibrosis, energy metabolism, and inflammation pathways. Then, we further investigated the affected genes in the significantly enriched pathways, and the results showed that fibrosis related genes such as *Tgfb1* and *Smad3* were significantly affected. Moreover, ERK/MAPK and TGF-β pathways were also dramatically enriched by STDP treatment (Supplementary Table S2), indicating that ERK/MAPK and TGF-β pathways might be the vital mechanisms of STDP against CHF. To better elucidate the biological significance of the DEGs, we performed functional enrichment analysis using Metascape. Interestingly, we found that MAPK cascade was similarly significantly enriched (Figure 5B). Therefore, we speculated that STDP might improve CHF mainly *via* the MAPK and TGF-β signaling pathways.

### STDP Exerted the Protective Effect Against CHF by Inhibiting ERK/MAPK and TGF-β Pathways

**FIGURE 6:**
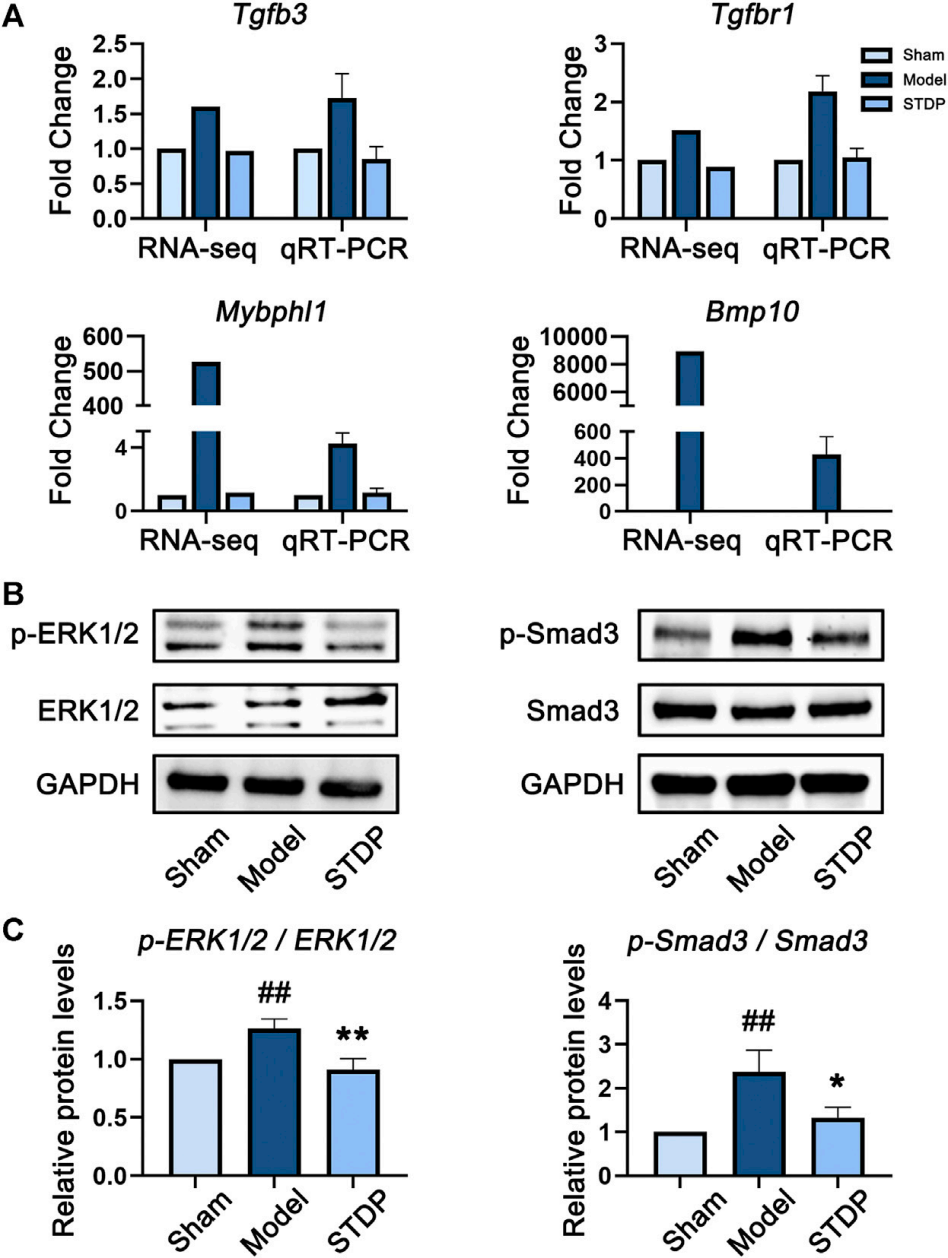
STDP protects mice against CHF *via* downregulating ERK/MAPK and TGF-β pathways. **(A)** The gene expression levels of *Tgfb3*, *Tgfbr1*, *Mybphl1*, and *Bmp10* detected by transcriptome sequencing and qRT-PCR. The internal reference was *Actb*, n = 3. **(B)** The protein expression levels of *p*-ERK1/2, ERK1/2, *p*-Smad3, and Smad3 in heart tissues detected by immunoblot analysis. GAPDH was used as the internal standard. **(C)** The quantitative results calculated by Image Lab software. ^##^ *p* < 0.01 versus the Sham group; ^*^ *p* < 0.05 and ^**^ *p* < 0.01 versus the Model group; n = 3.

Abundant evidence has indicated that the conduction of ERK/MAPK signaling pathway is closely related to myocardial fibrosis and hypertrophy (Sun et al., 2018), whereas TGF-β signaling pathway plays a key role in the occurrence and development of cardiac fibrosis (Yu et al., 2002; Leask, 2007). Therefore, according to the pathway enrichment results, we performed experimental verification on the expression of the key proteins in these two pathways in mouse heart tissues by Western blot analysis. The results were shown in Figures 6B,C. Specifically, compared with Sham group, TAC modeling conspicuously increased the phosphorylation level of Smad3 protein, while this trend was markedly reduced after STDP treatment (*p* < 0.01). These findings were consistent with the results of Masson’s trichrome staining, indicating that anti-myocardial fibrosis was one of the important mechanisms of STDP in improving CHF. In addition, a same manner was observed in the expression level of phosphorylated ERK1/2 protein, revealing that the protective effect of STDP on CHF might be related to the inhibition of ERK/MAPK pathway.

## Discussion

CHF is a severe and terminal stage of numerous heart diseases, caused by impaired cardiac relaxation and/or contraction (Lu et al., 2019). Despite advances have been made in the management and treatment of CHF in recent years, its mortality rate is still increasing annually (Bacmeister et al., 2019). To date, the mainstay of treatment for CHF remains pharmacotherapy (Ma and Liao, 2019). In view of the fact that the onset of CHF is associated with diversified factors, such as neurohormonal activation, ventricular remodeling, energy metabolism disorder, and increased hemodynamic overload (Tanai and Frantz, 2015), multi-target therapy may be more effective for the treatment of CHF.

In this study, we detected that STDP, a “multi-component, multi-target” drug, has anti-CHF effect *in vivo*. STDP can improve cardiac function, ameliorate myocardial fibrosis, and alleviate cardiomyocyte hypertrophy, showing a significant protective effect on TAC-induced CHF mice. Moreover, based on integrating network pharmacology and whole-transcriptome sequencing, our results demonstrated that STDP exhibits a global modulating of the multi-target activity in CHF disease network. Specifically, 395 CHF-related genes that were disturbed under TAC modeling presented an obvious recovery trend after STDP administration. Among them, there are 307 genes with a recovery rate greater than 80%, accounting for 77.7% of the total number of genes with the recovery trends. In addition, pathway analysis and *in vivo* validation experiment showed that modulation of fibrosis (the top1 enriched pathway), energy metabolism, and inflammation pathways may be the main mechanisms of STDP against CHF, and inhibition of ERK/MAPK and TGF-β signaling is the specific molecular events.

Accumulating evidence shows that impaired cellular energy production and transfer contribute to heart failure, in which mitochondrial dysfunction is the cause of energy deprivation (Brown et al., 2017). Moreover, mitochondrial fatty acid oxidation, the predominate mechanism to produce energy in the healthy adult human heart, declined in heart failure, whereas downregulation of peroxisome proliferator-activated receptor-α (PPARα) may be an important event leading to impaired mitochondrial fatty acid oxidation in this disease (Goikoetxea et al., 2006; Brown et al., 2017). Consistently, we detected that the energy metabolism-related pathways such as PPARα/RXRα activation and adipogenesis pathway were significantly enriched after STDP treatment (Supplementary Table S2), while the expression level of PPARα was obviously downregulated in model group and exhibited 87.61% efficiency of recovery regulation after STDP administration (Supplementary Table S3). Similarly, *Klf5*, encoding KLF5 protein which is a transcriptional regulator of PPARα (Drosatos et al., 2016), had a same expression trend after STDP treatment in CHF mice. Additionally, inflammation is associated with the progression of CHF and serves as one of the main therapeutic targets in patients with CHF (Amin et al., 2015). In this study, plenty of inflammation pathways were markedly enriched after STDP treatment, including neuroinflammation signaling and HMGB1 signaling, and inflammation-related genes such as *Tlr2*, *Tlr3*, *Ccl5*, *Il1b*, and *Il12b* were affected in these pathways (Supplementary Table S2). Previous study demonstrated that the Toll-like receptors are involved in the progression of CHF, whereas TLR2 can promote myocardial inflammation in heart failure (Yu and Feng, 2018). Increase of CCL5 serum levels was also observed in patients with severe congestive heart failure (Montecucco et al., 2012), whereas upregulation of IL-1β exhibited a significant increase in 1-year mortality in the patients with heart failure (Everett and Siddiqi, 2019). In this study, a recovery regulation of these genes (*Tlr2*, *Tlr3*, *Ccl5*, *Il1b*, and *Il12b*) was observed in the STDP-treated CHF mice (Supplementary Table S3), indicating anti-inflammation was also a key mechanism of SDTP against CHF.

Cardiac fibrosis, a common feature of myocardial injury, is caused by the imbalanced ratio of the collagen type I/III in the extracellular matrix (ECM) and excessive collagen deposition, which will lead to cardiac stiffness and cardiac insufficiency, and eventually cause CHF (Shababi et al., 2012; Xu et al., 2021). Consequently, inhibition of the key pro-fibrotic stimuli is considered as a potential strategy against CHF. TGF-β is the master factor in cardiac fibrosis under pressure overload situation (Meng et al., 2016; Pardali et al., 2017; Yao et al., 2020), while Smad3 is a target protein for TGF-β signal transduction and mediates the transport of TGF-β signals from cell membrane receptors to the nucleus promoting the transcription of various genes that regulate fibrosis (Flanders, 2004). Evidences indicate that inhibition of TGF-β/Smad signaling plays a critical role in restraining cardiac fibrosis (Buxton and Duan, 2008; Lu et al., 2019; Wang et al., 2020). In line with this, using a mouse model of pressure overload-induced heart failure by TAC, we discovered that TGF-β pathway was significantly deregulated and the phosphorylation of Smad3 was almost completely blocked after STDP treatment, suggesting inhibiting the TGF-β/Smad signaling is mainly involved in the anti-cardiac fibrosis effects of STDP on CHF mice. Consistently, in-depth analysis of the affected genes in the significantly enriched pathways revealed that TGF-β1 and Smad3 appeared in multiple signaling pathways (Supplementary Table S2), such as cardiac hypertrophy signaling and neuroinflammation signaling pathway. MAPK is also known to play important roles in cardiac development, pathological ventricular remodeling, and cardiac hypertrophy (Rose et al., 2010). MAPK signaling cascade is associated with TAC-induced cardiac hypertrophy (Sun et al., 2018). After TAC surgery, ERK, JNK, and p38 MAPK are simultaneously activated, leading to cardiac hypertrophy and, subsequently, CHF (Liu et al., 2016). ERK is a vital participant in the pathophysiology of myocardial hypertrophy (Gallo et al., 2019). In contrast to ERK, however, the role of JNK is blurred in cardiac hypertrophy (Sun et al., 2018). Our study confirmed that 7 weeks after TAC modeling, ERK1/2 phosphorylation was significantly increased, while STDP treatment completely blocked the phosphorylation of ERK1/2 in cardiac tissue, resulting reduction in the size of hypertrophic cardiomyocytes. Similar effects were also found in the main constituents of STDP, such as gentisic acid, taurine, and tanshinone IIA (Shi et al., 2002; Zhan et al., 2014; Sun et al., 2018). Specifically, gentisic acid reduced TAC-induced cardiac hypertrophy and myocardial fibrosis in mice by inhibiting the ERK1/2 signaling pathway. Taurine could diminish the damage associated with oxidative stress and calcium overload (Shiny et al., 2005), and prevent AngII-mediated cardiac fibrosis (Takahashi et al., 1997), showing a beneficial effect in the treatment of CHF (Ito et al., 2014). tanshinone IIA inhibited myocardial fibrosis by inhibiting TGF-β1-mediated phosphorylation of Smad2/3. Besides, we also established a network between the STDP main constituents and the targets which presented a recovery trend after STDP treatment (Supplementary Figure S2A). Thirteen constituents were included due to their high content or according to the literature they had beneficial effects in ameliorating CHF symptoms (Chen et al., 2015), *i.e.,* gentisic acid, taurine, tanshinone IIA, danshensu, protocatechualdehyde, salvianolic acid D, salvianolic acid B, salvianolic acid A, taurodeoxycholic acid, cinobufotalin, bufalin, cinobufagin, and resibufogenin. After prediction, 169 potential targets were obtained using PharmMapper database. An intersection between these potential targets (169 genes) and the genes with the recovery efficiency EoR >0 (395 genes) after STDP treatment was generated. As a result, 28 genes were induced in common and a “compound-target” network was constructed to this gene set using CytoscapeV3.8.0. Pathway enrichment analysis further suggested that these potential targets were mainly involved in the chemokine signaling, inflammation, atherosclerosis, and renin-angiotensin system (Supplementary Figure S2B). Since these pathways are highly related to CHF progression and the expression of these targets was obviously recovered after STDP treatment, it indicated that these compounds may be the potential effective constituents of STDP against CHF.

In addition to the key targets mentioned above, it was worth noting that STDP may also act on several new targets for anti-heart failure. BRD4 (Bromodomain-containing protein 4), a member of the bromodomain and extra terminal protein family, has been confirmed to play an important role in improving heart failure (Duan et al., 2017; Li et al., 2020b). Inhibition of BRD4 could block agonist-induced pathological hypertrophy in human induced pluripotent stem cell-derived cardiomyocytes, and attenuate TAC-induced myocardial fibrosis in mice (Duan et al., 2017; Song et al., 2019). High expression of Wnt5a (Wnt family member 5a) may promote myocardial inflammation and fibrosis, thereby contributing to heart failure progression (Abraityte et al., 2017a). A recent study reported that Wnt5a was significantly increased in the serum and myocardium of patients with heart failure and decreased after left ventricular assist device therapy (Abraityte et al., 2017b), implying that Wnt5a may be an ideal molecular target for CHF treatment. CREB1 (cAMP responsive element-binding protein 1) had been implicated in the pathophysiology of heart failure (Duan et al., 2017). Downregulating the CREB1-mediated circ-HIPK3 expression level could reduce the degree of fibrosis after myocardial infarction and maintain cardiac function (Deng et al., 2019). Furthermore, serum syndecan-4 (SDC4) may develop as a novel diagnostic biomarker and therapeutic target in patients with CHF (Takahashi et al., 2011), showing its great clinical potential for CHF. Interestingly, in this study, the expression levels of these genes (BRD4, Wnt5a, CREB1, SDC4) were all upregulated after TAC surgery, while STDP treatment significantly reversed this trend. The efficiency of recovery regulation for these genes was 99.85% (BRD4), 99.79% (Wnt5a), 99.8% (CREB1), and 99.8% (SDC4), respectively (Supplementary Table S3; BRD4 is not shown in Supplementary Table S3 because it was only present in the sequencing data, but this gene had an obvious recovery regulation after STDP treatment in CHF mice). Taken together, these results illustrated that STDP shows potential as a possible new drug for treatment of CHF.

## Conclusion

In summary, the present study demonstrated that the protective effects of STDP on CHF was mainly reflected by improving cardiac function, inhibiting cardiomyocyte hypertrophy, and anti-myocardial fibrosis in mice. Moreover, the key mechanism of STDP against CHF was revealed by combining network pharmacology analysis with whole-transcriptome sequencing, which was related to modulate critical pathways in disease network, such as fibrosis, energy metabolism, and inflammation pathways. Additionally, the present study demonstrated that reducing myocardial fibrosis and cardiomyocyte hypertrophy by inhibition of TGF-β and ERK/MAPK signaling pathways may be the key pharmacological mechanism of STDP in ameliorating CHF. Taken together, our results suggested that STDP shows great potential for the treatment of CHF and is hopeful to be developed as a new drug for CHF.

## Data Availability

The original contributions presented in the study are publicly available. This data can be found here: https://www.ncbi.nlm.nih.gov/geo/query/acc.cgi?acc=GSE185631.
