# Supplementary material for: Shexiang Tongxin Dropping Pill Protects Against Chronic Heart Failure in Mice via Inhibiting the ERK/MAPK and TGF-β Signaling Pathways
Source: Front Pharmacol. 2021 Dec 3;12:796354. doi: 10.3389/fphar.2021.796354 (PMC8682969; doi:10.3389/fphar.2021.796354)
Supplement: Supplementary file 1 [file DataSheet1.docx]

Supplementary Material

# Supplementary Methods

## HPLC analysis of STDP

STPD samples (40 pills) were ground into fine powder without the film coating and thoroughly mixed. Subsequently, STDP powder was accurately weighed, extracted with 10 mL methanol in an ultrasonic water bath at room temperature for 30 min, and the extract was filtered (0.22 µm, Millex-GN Nylon, Millipore). The filtrate was collected and analyzed for HPLC analysis. The HPLC analysis was performed on an Agilent HPLC 1260 series instrument (Agilent, Waldbronn, Germany), equipped with an auto sampler, a binary pump, a thermostatic column compartment, a degasser, and a DAD. The separation was carried out on a Synergi^TM^ Hydro-RP column (250 mm × 4.6 mm, 4 µm particle size). The mobile phase was composed of acetonitrile with 0.02% phosphoric acid (A) and water with 0.02% phosphoric acid (B) according to pre-set gradient program: 0 to 5 min, 5% (A), 95% (B); 5 to 13 min, linear gradient 5% to 20% (A), 95% to 80% (B); 13 to 40 min, linear gradient 20% to 22% (A), 80% to 78% (B); 40 to 60 min, linear gradient 22% to 45% (A), 78% to 55% (B); 60 to 75 min, linear gradient 45% to 70% (A), 55% to 30% (B). The column temperature was 30°C, the injection volume was 10 μL, and the flow rate was 1.3 mL/min. The detection wavelength was set to 220 nm. The HPLC chromatograms of STDP was recorded and the main peaks were identified by comparing with reference standards.

## Network construction and analysis using the main constituents and the targets which presented a recovery trend after STDP treatment.

The molecular structures of 13 main constituents of STDP (*i.e.,* gentisic acid, taurine, tanshinone IIA, danshensu, protocatechualdehyde, salvianolic acid D, salvianolic acid B, salvianolic acid A, taurodeoxycholic acid, cinobufotalin, bufalin, cinobufagin, and resibufogenin) were first collected using PubChem (https://pubchem.ncbi.nlm.nih.gov/) and Traditional Chinese Medicine Systems Pharmacology (TCMSP, https://old.tcmsp-e.com/tcmsp.php) and further imported into PharmMapper (http://lilab-ecust.cn/pharmmapper/) to predict the potential targets. Then, the Uniprot Retrieve/ID mapping tool (https://www.uniprot.org/uploadlists/) was used to convert the UniProtKB AC/ID to Gene Name. Next, the intersection of these potential targets (169 genes) and the genes with the recovery efficiency EoR > 0 (395 genes) after STDP treatment was obtained and 28 genes were induced in common. Finally, the protein-protein interactions (PPI) analysis and KEGG enrichment analysis was performed on these 28 genes in the String database (https://string-db.org/), and the network visualization was conducted using the CytoscapeV3.8.0 software.

**
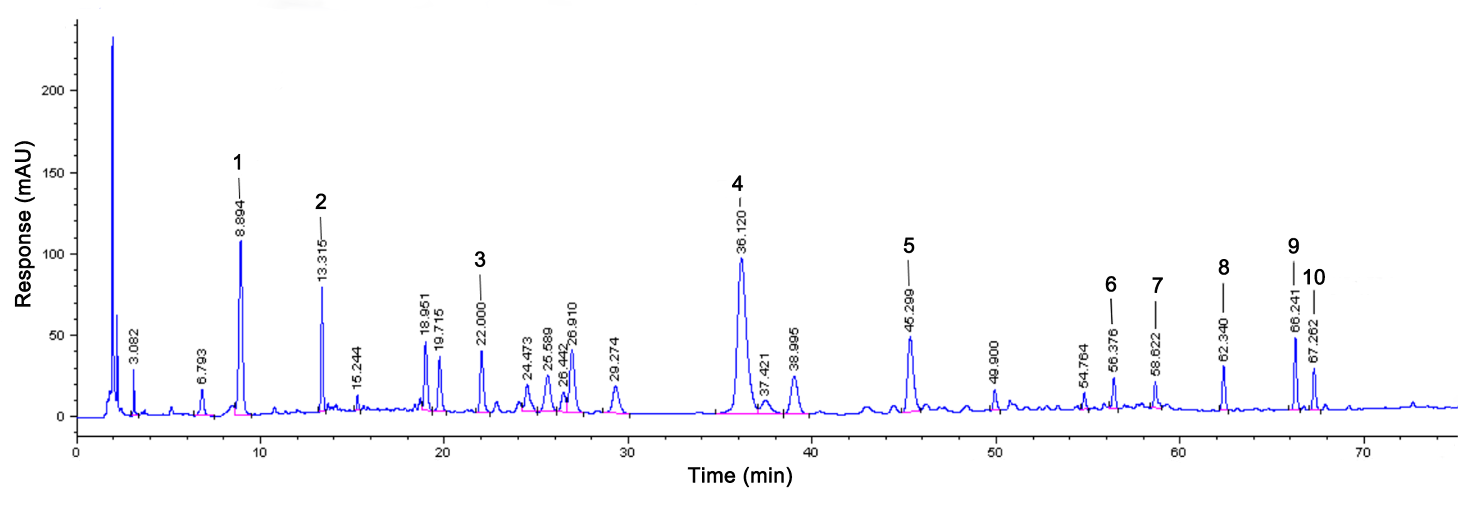
**

**Supplementary Fig.1** Representative HPLC chromatogram of STDP. Ten chromatographic peaks were identified: (1) danshensu, (2) protocatechualdehyde, (3) salvianolic acid D, (4) salvianolic acid B, (5) salvianolic acid A, (6) taurodeoxycholic acid, (7) cinobufotalin, (8) bufalin, (9) cinobufagin, (10) resibufogenin.


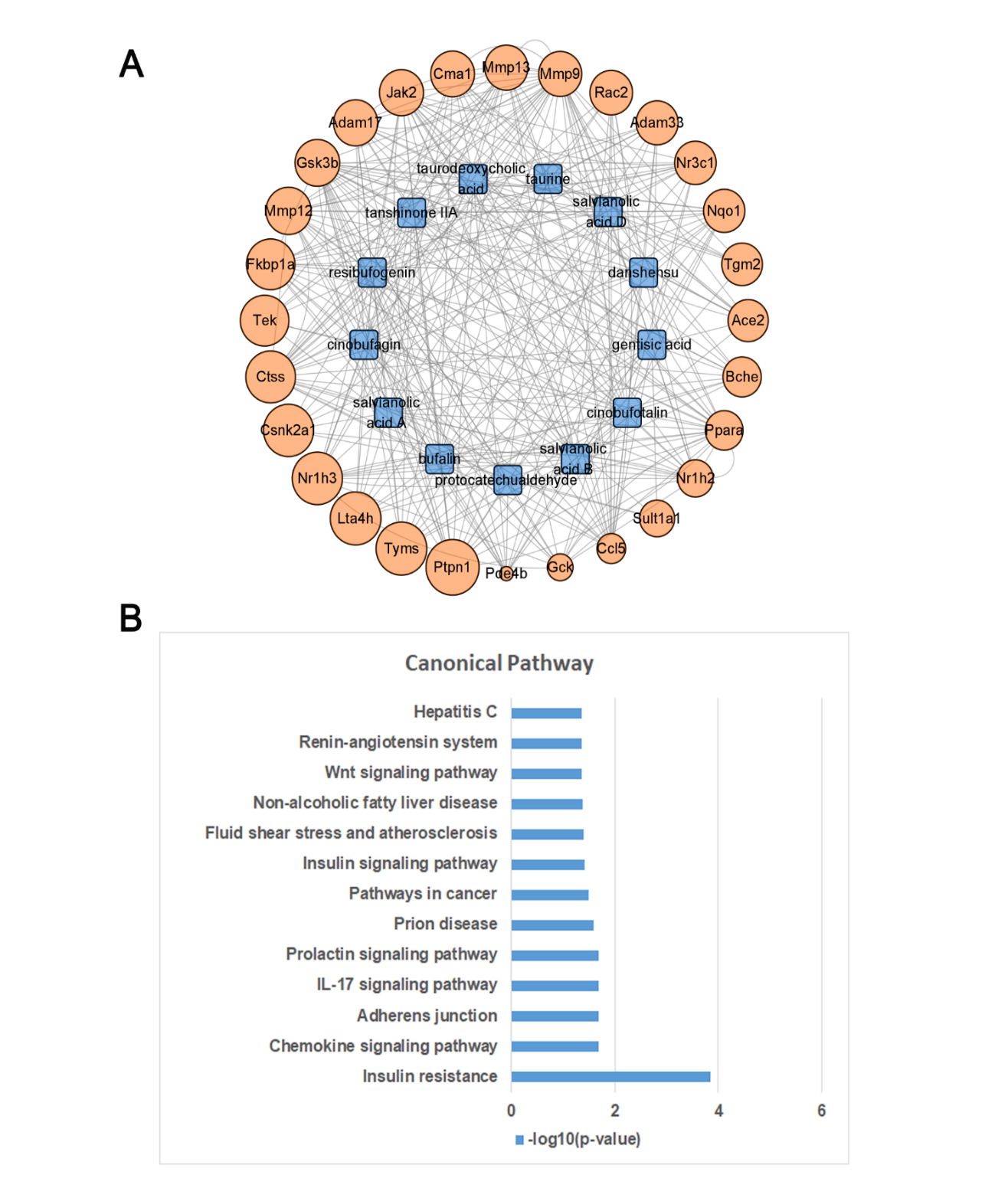


**Supplementary Fig. 2** Network construction and analysis using the main constituents and the targets which presented a recovery trend after STDP treatment. (A) The compound-target network was established using Cytoscape 3.8.0 software. The blue squares represent the main constituents of STDP. The orange circles indicate the potential targets of CHF regulated by the main bioactive compounds of STDP with the recovery efficiency EoR > 0. The size of orange circles represents the efficiency of recovery regulation. (B) The KEGG pathway enrichment analysis of the 28 potential targets of STDP main constituents.
